# Potential Therapeutic Targets of Quercetin and Its Derivatives: Its Role in the Therapy of Cognitive Impairment

**DOI:** 10.3390/jcm8111789

**Published:** 2019-10-25

**Authors:** Md. Jakaria, Shofiul Azam, Song-Hee Jo, In-Su Kim, Raju Dash, Dong-Kug Choi

**Affiliations:** 1 Department of Applied Life Sciences and Integrated Bioscience, Graduate School, Konkuk University, Chungju 27478, Korea; 2 The Florey Institute of Neuroscience and Mental Health, The University of Melbourne, Parkville, Victoria 3010, Australia; 3 Department of Integrated Bioscience and Biotechnology, College of Biomedical and Health Sciences, and Research Institute of Inflammatory Diseases (RID), Konkuk University, Chungju 27478, Korea; 4 Department of Anatomy, Dongguk University Graduate School of Medicine, Gyeongju 38066, Korea

**Keywords:** quercetin, nervous system, molecular signals, pharmacological potential, cognitive impairment

## Abstract

Quercetin (QC) is a flavonoid and crucial bioactive compound found in a variety of vegetables and fruits. In preclinical studies, QC has demonstrated broad activity against several diseases and disorders. According to recent investigations, QC is a potential therapeutic candidate for the treatment of nervous system illnesses because of its protective role against oxidative damage and neuroinflammation. QC acts on several molecular signals, including ion channels, neuroreceptors, and inflammatory receptor signaling, and it also regulates neurotrophic and anti-oxidative signaling molecules. While the study of QC in neurological disorders has focused on numerous target molecules, the role of QC on certain molecular targets such as G-protein coupled and nuclear receptors remains to be investigated. Our analysis presents several molecular targets of QC and its derivatives that demonstrate the pharmacological potential against cognitive impairment. Consequently, this article may guide future studies using QC and its analogs on specific signaling molecules. Finding new molecular targets of QC and its analogs may ultimately assist in the treatment of cognitive impairment.

## 1. Introduction

Cognitive impairment is very common in various neurological disorders, which affect the thinking, communication, understanding, and memory of a person. A patient’s cognitive function might be affected in several neurodegenerative diseases (NDDs) such as Alzheimer’s disease (AD), Parkinson’s disease (PD), multiple sclerosis, or stroke [1]. The common pathological characterization of these disorders is one of the progressive dysfunctions and neuronal injury, leading to a slow and irreversible deterioration in brain function. These multifactorial and debilitating disorders affect approximately 30 million individuals worldwide [2].

Due to the complexity in the mechanistic progression of cognitive impaired disorders, illuminating the proper disease pathophysiology and therapeutics of cognitive impairment remains a foremost challenge [3]. No curative treatment for cognitive impairment currently exists, an alternative would be to find ways to attenuate cognitive impairment in older people, which, in turn, could delay the onset of cognitive impairment [4]. Numerous phytochemicals have received significant attention as potential agents in treating neurodegenerative conditions, as adjuncts to modern medicines [1,5,6,7]. Quercetin (QC), a readily available natural polyphenol, is one such phyotochemical, which is abundant in vegetables and fruits, and considered to be the main flavonoid in our daily diet [8]. QC displays broad spectrum properties against inflammation and cancer [9]. It is also a crucial bioactive compound, protective against injuries to the nervous, hepatic, cardiovascular, and urinary systems [10,11,12]. In a recent study on a mild traumatic brain injury (TBI)-induced mouse model, the treatment with QC significantly reduced anxiety-like behaviors of mice. QC treatment also ameliorated the dysregulation of the hypothalamic–pituitary–adrenal axis in TBI-induced mice and decreased levels of adrenocorticotropic hormones and corticosterones [13]. The protective role of QC against cognitive impairment has been demonstrated in several studies [9,10,14], just as recent advances have led to an increased understanding of the processes underlying cognitive impairment [15]. The identification of molecules that contribute to the pathological progression of disease is crucial in the therapy and drug discovery process, therefore, in this article, we discuss several potential targets where QC displays pharmacological activity against cognitive impairment along with an overview of QC chemistry and biopharmaceutics.

## 2. Overview of Quercetin

Among the over 4000 naturally occurring phenolic compounds in plants, QC was isolated and defined as biologically active by Szent-Gyorgyi in 1936 [16]. QC (5,7,3′,4′-hydroxyflavonol) is found in onions, curly kale, leeks, broccoli, apples, tea, capers, and blueberries, with onion (as 300 mg/kg of fresh onion) often contributing the most to total QC intake [17]. QC is a promising dietary component in the prevention of lifestyle diseases due to its wide-ranging effects [18]. In addition, we have published that methanol extract of red onion protects against lipopolysaccharide (LPS) and 1-methyl-4-phenylpyridinium, and upregulates the antioxidant enzymes that could potentially be used in the therapy of NDDs [19].

**Figure 1:**
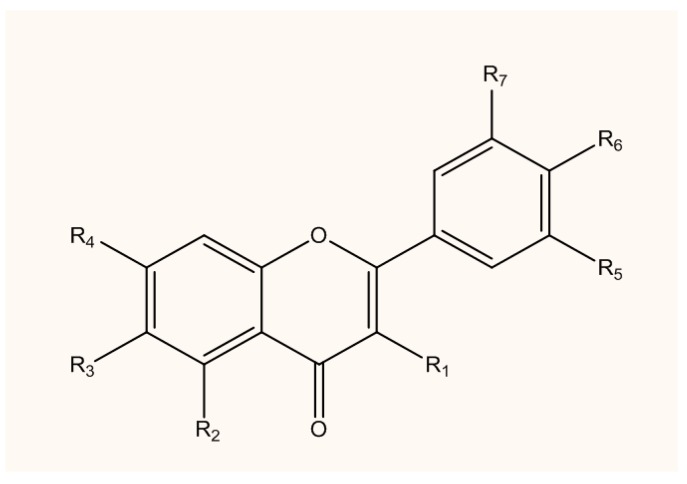
The chemical structure of quercetin (QC). The changing of functional groups makes derivatives of QC.

QC is naturally available as derivatives either in glycosidic form (primarily bound to glucose and rutinose), bound to ethers, and, very rarely, as a sulfate and prenyl substituent [20,21]. QC O-glycosides are QC derivatives with at least one O-glycosidic bond primarily glycosylated at the hydroxyl group of C-3 carbon with galactose, glucose, xylose or rhamnose [10]. The backbone of the QC chemical structure (Figure 1) and QC derivatives, with their potential targets for neuroprotection are listed in Table 1 [22]. Another glycosylation site is the hydroxyl group of C-7 carbon, for example, QC 7-O-glucoside, which is accessible in beans [23]. Ether derivatives of QC are formed between the OH group of QC and an alcohol molecule, typically methanol. QC ether derivatives are connected to sugar moiety groups, including 7-methoxy-3-glucoside and QC 30-methoxy-3-galactoside, which occurs widely in nature [24]. The presence of the five-hydroxyl group makes QC a highly lipophilic compound, although solubility of QC-derivatives is dependent on the type of substituent molecules existing in the OH group. C-methyl, O-methyl and prenyl derivatives of QC are lipophilic in nature [21]. Glycosylation of QC raises hydrophilicity, and these glycosylated derivatives are cytosol soluble, simply transported to all fragments of the plants and frequently deposited in vacuoles [21,25].

The unique structure of QC allows it to demonstrate a potent antioxidant action. The functional groups of QC are accountable for the stability and antioxidant activity; these are 3- and 5-OH groups, in conjugation with the 4-oxo group and the orthodihydroxy or catechol group [26]. QC gives a proton to free radicals, for example, 2,2-diphenyl-1-picrylhydrazyl and converts itself into a quinone intermediate, which is steadied by the electrons donated by these functional groups [27]. QC derivatives such as C3 and C4__OH glycoside derivatives display reduced H- donating capability. The decreasing potential of C3__OH derivatives of QC is greater when compared to its aglycone form [28].

Aglycone QC bioavailability is poor, and in human plasma, free QC has been not found after oral ingestion of QC [29]. As stated earlier, QC occurs in the form of glycosides in fruits and vegetables. Upon dietetic intake, QC glycosides are quickly hydrolyzed in the epithelial cells, with the help of β-glucosidase enzyme or by bacterial action in the colon, to make QC aglycone, which is readily absorbed in the large intestine. QC aglycone is then transported to the liver via portal circulation, where it starts glucuronidation, O-methylation, and/or sulphation to form its conjugates QC-3-glucuronide, QC-30-sulphate, and iso-rhamnetin-3-glucuronide [26,30,31]. The nature and binding site of glycosides at the position of 3, 5, 7, or 40 determine the extent of absorption. Wittig et al. [32] displayed that fried onion consumption exhibited only the presence of QC glucuronides and not QC aglycone or glucoside, which designates that the conjugated form of QC is more extensively found in plasma than its aglycone form. QC intake with fat-enriched diets and emulsifiers elevated the QC metabolite accumulation in blood plasma by suggestively delaying its excretion [33].

Metabolites of QC follow two dissimilar approaches of excretion such as part of biliary secretions into the small intestine or the urine [34]. Any QC absorption in the small intestine appears futile, as it is degraded by microflora, along with that secreted in the bile; the subsequent aglycone endures ring fission, leading to hydroxycinnamates and phenolic acid production [35]. QC metabolites follow a slow elimination rate (the range of half-lives are 11 to 28 h) [33]. A lower dose of QC can be more methylated than higher doses in humans [36]. Additionally, sulphation is usually a greater affinity, lower capacity pathway than glucuronidation; an elevate in the amount of QC swallowed may lead to a shift from sulphation toward glucuronidation. An extensive amount of research utilizing QC aglycone in vitro has been done due to the very low concentrations of aglycone found in plasma.

Harwood et al. have extensively reviewed the safety of QC [37]. Briefly, QC is not categorized as mutagenic or carcinogenic in vivo [38], albeit it has been found to hinder CYP3A4, an enzyme that breaks down numerous frequently prescribed drugs [39]. Thus, QC should not be ingested in combination with drugs including colchicine and alprazolam (Xanax), which depend on this pathway for appropriate metabolism [40].

The dietary intake of all flavonoids has been estimated to be approximately 200–350 mg/d, and that of flavonols, approximately 20 mg/d, with QC accounting for nearly 50% of this total (given a daily intake of approximately 10 mg/d) [41]. The results of a Japanese study reinforced these approximations, as daily intake of QC was determined to be 16 mg [19]. QC aglycone is sold as a nutritional supplement, with a suggested dosage of 1 g/d [37]. Over-the-counter supplemental QC is available in 250 and 1500 mg capsules and is marketed as being helpful for a variety of ailments such as eye disorders, asthma, gout, arthritis, allergies, bacterial infections, hypertension, and neurodegenerative disorders [42].

## 3. Potential Therapeutic Targets of Quercetin

### 3.1. Voltage-Gated Ion Channels

Several studies have demonstrated the potential role of QC action on voltage-gated ion channels. In one such study, the polyphenols QC, catechin, and resveratrol from red grapes were found to prevent peak INa, with half maximal inhibitory concentration (IC50s) of 19.4, 76.8, and 77.3 μM, correspondingly. Resveratrol and QC reduced the voltage-gated sodium channel (VGSC) long QT mutant R1623Q-induced late INa. Resveratrol and QC also blocked anemonia viridis toxin 2 (ATX II)-induced late INa, with IC50s of 26.1 μM and 24.9 μM, correspondingly. The inhibitory action of QC on cardiac VGSCs may thus potentially contribute to the cardioprotective effectiveness of products containing red grape extract [61].

QC may hold potential as an agent to treat cerebral ischemia and vascular dementia due to its inhibitory effect on the sodium channel [62]. In a study conducted on chronic cerebral ischemia in rats, QC (5 mg/kg i.p. for 14 d) improved the cognitive performance of ischemic rats on the Morris water maze test. In electrophysiological experiments, QC attenuated the prevention of long-term potentiation in ischemic rats. Also, in acutely isolated rat hippocampal CA1 pyramidal neurons, QC (0.3, 3.0, and 30.0 μM) reduced the amplitude of voltage-dependent sodium currents in a voltage- and dose-dependent way [62].

QC affects the release of glutamate in rat cerebral cortex nerve terminals (synaptosomes). Treatment with QC prevented glutamate release evoked by 4-aminopyridine (4-AP), which is a K+ channel blocker but chelating extracellular Ca^2+^ ions inhibited this effect. QC reduced the depolarization-induced elevate in the cytosolic free Ca^2+^, while it did not alter 4-AP-mediated depolarization and Na+ influx. The QC-mediated prevention of glutamate release was reversed by antagonizing the Cav2.2 (N-type) and Cav2.1 (P/Q-type) channels. The combined prevention of protein kinase C (PKC) and protein kinase A (PKA) also blocked the antagonistic action of QC on evoked release of glutamate. In addition, QC declined the 4-AP-induced phosphorylation of PKC and PKA. As per these findings that QC-mediated inhibitory action on glutamate release from rat cortical synaptosomes is connected both to a decrease in presynaptic voltage-dependent entry of Ca^2+^ and to the prevention of the activity of PKC and PKA [63].

QC protects against bupivacaine-induced neurotoxicity in SH-SY5Y cells. Treatment with QC (50 μM) significantly prevented bupivacaine-intoxicated cell apoptosis and declined intracellular Ca^2+^ concentration in SH-SY5Y cells. QC treatment also normalized Cav3.1 protein expression. Thus, QC-treatment decreased bupivacaine-intoxicated toxicity, probably via inhibition of the T-type calcium channel. QC-mediated this action may indicate its potential in the treatment of local anesthetic agent-mediated toxicity [64].

### 3.2. Neuroreceptors

#### 3.2.1. Dopamine Receptors

QC displayed protective activity in several models of toxic agent-induced PD. In a 6-hydroxydopamine (6-OHDA)-intoxicated PD model, QC showed neuroprotective activity by displaying effects against oxidative stress [65,66]. The treatment with QC (30 mg/kg body weight, over 14 days) markedly elevated the glutathione and striatal dopamine levels compared with 6-OHDA-induced group [65]. QC also showed protective activity against 1-methyl-4-phenyl-1,2,3,6-tetrahydropyridine (MPTP)-induced PD-like syndromes, where it prevents MPTP-induced loss of dopamine in the mice brain [67]. In addition to 6-OHDA and MPTP, QC treatment also ameliorated acrylamide-induced memory impairment by increasing dopamine content [49]. While QC showed protective activity in PD models, only a few studies have investigated its effect on dopamine receptors. In one study, the D2 agonist quinpirole (0.2 mg/kg) potentiated QC (200 mg/kg) antinociceptive activity, but dopamine D1 receptor agonist, SKF38393 (10 and 15 mg/kg), was unsuccessful in modifying the QC-mediated antinociceptive effect. QC (200 mg/kg) prevented reserpine-intoxicated (2 mg/kg, 4 h) hyperalgesia, which was inverted by sulpiride. Thus, a role of alpha2-adrenoreceptors and dopamine D2 receptors is hypothesized in the antinociceptive effect of QC [68].

In a polychlorinated biphenyl (PCB/Aroclor-1254)-induced rat model, QC ameliorated against PCB-treated impairment of dopaminergic receptor expressions in the hippocampus [69]. Upon PCB induction, hydrogen peroxide was generated, and lipid peroxidation was induced in the hippocampus, which led to a disturbance of ATPases and dopamine receptor expressions [69]. PCB also caused an alteration in the expression of tyrosine hydroxylase (TH). Changes in the dopaminergic receptor expressions at mRNA and protein levels evidently represent the adverse role of PCBs on the dopaminergic system, which may, in turn, influence cognitive impairment. The reduction in calcium voltage-gated channel subunit alpha1 D gene expression indicates there may also be an alteration in neurotransmitter release and signal transduction [69]. Administering QC reinstated the biochemical and morphological changes in the PCB-intoxicated hippocampus [69].

The exposure of cadmium in rat corpus striatum and PC12 cells leads to a selective reduction in dopamine (DA)-D2 receptors, which affected the post-synaptic PKA/ protein phosphatase 1 regulatory subunit 1B/type 1 protein phosphatase alpha and β arrestin/protein kinase B (Akt)/glycogen synthase kinase 3β signaling concomitantly. The antagonism of PKA and c in vitro reveals that both pathways are independently moderated by DA-D2 receptors and are connected to the cadmium-induced motor abnormalities. Ultrastructural deviations in the corpus striatum confirmed neuronal collapse and loss of synapses on cadmium induction. According to the molecular docking hypothesis, the direct interaction of cadmium with the dopamine on DA-D2 receptor competitive sites may be connected to the decrease in DA-D2 receptors. As treatment with QC ameliorated cadmium-induced behavioral and neurochemical variations, QC may be a potential agent to ameliorate cadmium-induced dopaminergic dysfunctions [70].

#### 3.2.2. Glutaminergic Receptors

QC acts on ionotropic glutamate receptors, and it is reported to exhibit neuroprotective activity in a dexamethasone-induced cognitive deficit mouse model. The administration of dexamethasone changed the expression of N-methyl-D-aspartate (NMDA) receptors in the hippocampus, while pretreatment with QC protected against a reduction in NMDA receptor expression [71]. In addition, QC pretreatment demonstrated antidepressant activity in an olfactory bulbectomy mouse model that involved the NMDA receptor; the administration of NMDA reversed QC-mediated antidepressant activity [72]. According to the computational study, QC may be a potential ligand of the α-amino-3-hydroxy-5-methyl-4-isoxazolepropionic acid (AMPA) receptor as it shows a superior docking score [73]. QC action on the glutamate-treated inward current (IGlu) in Xenopus oocytes that heterologously express human AMPA receptor and stargazin, was examined. The two-electrode voltage clamp technique was employed to measure IGlu. In oocytes inserted with cRNAs coding for AMPA receptor (GluA1) and stargazin, QC-mediated prevention of IGlu was in a concentration- and reversible-dependent manner. The activity of QC on IGlu was ameliorated by elevating glutamate concentration, and the activity was membrane holding potential dependent. These findings indicate that QC interacts with the AMPA receptor, which was heterologously expressed in Xenopus oocytes. The action of QC on the IGlu of the AMPA receptor may show potential for neuroprotective therapy [73]. In a recent study, QC ameliorated kainic acid (KA)-induced seizures in mice. Pretreatment with QC (100 mg/kg) significantly elevated gene expression of the GluA1 subunit of AMPA and the GluN2A and GluN2B subunits of NMDA only 7 d after KA intoxication, in comparison with the control and KA groups. Enhancement in the QC-mediated gene expressions of AMPA and NMDA receptor subunits may be indicating its protective effect on the synaptic plasticity and memory [74].

#### 3.2.3. Acetylcholine Receptors

Several studies have revealed QC’s actions on nicotinic acetylcholine receptors. In a study on α9α10 nicotinic acetylcholine receptor-dependent ion currents, the action of QC was examined utilizing the two-electrode voltage clamp technique. The treatment with acetylcholine evoked inward currents (IACh) in oocytes heterologously expressing the α9α10 nicotinic acetylcholine receptor. IACh was prevented by QC treatment in a concentration-dependent and reversible manner. The pre-application of QC on IACh was stronger than its co-application, and the IC50 of QC was 45.4 ± 10.1 μM. QC-mediated prevention of IACh was not affected by the concentration of acetylcholine and was independent of membrane-holding potential. While the preventive action of QC was significantly weakened in the absence of extracellular Ca^2+^, the QC effect was independent of extracellular Ca^2+^ concentration, suggesting that extracellular Ca^2+^ availability might be required for QC-mediated action and might play a critical role in QC-treated α9α10 nicotinic acetylcholine receptor regulation [75].

In another study, the treatment with acetylcholine elicited an IACh in oocytes expressing both muscle types of nicotinic acetylcholine receptors. QC cotreatment with acetylcholine protected IACh. QC pretreatment further protected IACh in oocytes expressing adult and fetal muscle-type nicotinic acetylcholine receptors. IACh prevention by QC was reversible and concentration-dependent, and the IC50 of QC was 18.9 ± 1.2 µM in oocytes expressing adult muscle-type nicotinic acetylcholine receptors. The prevention of IACh by QC was voltage independent and noncompetitive. According to these results, QC may have the potential to regulate the action of human muscle-type nicotinic acetylcholine receptors. QC-treated muscle-type nicotinic acetylcholine receptor regulation might be coupled with the regulation of neuromuscular junction action [76].

The actions of QC on heteromeric neuronal α3β4 nicotinic acetylcholine receptor channel action expressed in Xenopus oocytes after injection of cRNA encoding bovine neuronal α3 and β4 subunits have also been studied. Acetylcholine treatment provoked an IACh in oocytes expressing α3β4 nicotinic acetylcholine receptors, while cotreatment with QC and acetylcholine prevented IACh in oocytes expressing α3β4 nicotinic acetylcholine receptors. The prevention of IACh by QC was in a concentration-dependent and reversible manner. In oocytes expressing the α3β4 nicotinic acetylcholine receptor, the IC50 of QC was 14.9 ± 0.8 µM, and the antagonism of IACh by QC was voltage independent and noncompetitive. Therefore, QC might control the α3β4 nicotinic acetylcholine receptor, which may have pharmacological importance in the treatment of nervous system disorders [77].

It has been found that QC can elevate α7 nicotinic acetylcholine receptor (α7 nAChR)-dependent ion currents [78]. The action of QC glycosides on the acetylcholine-mediated peak IACh in Xenopus oocytes expressing the α7 nAChR has been studied. In oocytes injected with α7 nAChR copy RNA, QC increased IACh, while QC glycosides prevented IACh. As QC glycosides mediated inhibition of IACh, the mediation effect increased when the QC glycosides were pre-applied, and the preventive actions were concentration dependent. The order of IACh prevention by QC glycosides was Rutin≥Rham1>Rham2. QC glycoside-mediated IACh improvement was not affected by ACh concentration and appeared to be voltage independent. Additionally, QC-mediated IACh prevention can be ameliorated when QC is co-treated with Rham1 and rutin, demonstrating that QC glycosides could interfere with QC-mediated α7 nAChR regulation and that the carbohydrate numbers in the QC glycoside plays a crucial role in the disruption of QC-mediated effect. Thus, QC and QC glycosides control the α7 nAChR in a differential manner [79].

In a 2014 study, muscarinic acetylcholine receptor-active compounds were shown to have potential use in the treatment of AD [80]. As this computational study demonstrated the interaction of QC with the M1 muscarinic acetylcholine receptor [80], QC may represent a possible agent in the treatment of the disease.

#### 3.2.4. Serotonergic Receptors

A few studies have addressed the action of QC on serotonin receptors. In one such study in 2014, QC administered with ascorbic acid was found to prevent monoamine oxidase-A activity in SH-SY5Y cells by targeting mitochondria [81]. This combination also employed operative vasodilator actions in isolated pulmonary artery and prevented proliferation of cells and induced apoptosis in human pulmonary artery smooth muscle cells. QC-treated, these actions were connected to the decrease in expression of serotonin 2A receptor (5-HT_2A_) receptor and Akt and S6 phosphorylation and partly restored Kv currents. Thus, QC could be valuable in the management of pulmonary hypertension [82]. In oocytes injected with 5-HT_3A_ receptor cRNA, QC inhibited the 5-HT-treated inward peak current (IC50: 64.7 ± 2.2 μM) in a competitive and voltage-dependent manner. QC cooperates with the pre-transmembrane domain 1 (pre-TM1) of the 5-HT3A receptor because point mutations of pre-transmembrane domain 1 (pre-TM1) including R222T and R222A—but not R222D, R222E, and R222K—abolished prevention [83].

#### 3.2.5. Gamma-aminobutyric acid-ergic Receptors

Several investigations have focused on the anxiolytic activity of QC. For example, in a behavioral study using a mouse model, QC displayed significant anxiolytic activity. A gamma-aminobutyric acid (GABA)A-ρ agonist (trans-4-aminocrotonic acid, 20 mg/kg) antagonized the anxiolytic-like activities of QC. On the other hand, WAY-100635 (a 5-HT1A antagonist, 0.3 mg/kg) and flumazenil (a GABAA antagonist, 10 mg/kg) did not antagonize a QC-mediated anxiolytic effect. Therefore, the promoting anxiolytic-like activity of QC may be mediated by the GABAergic nervous system [84]. In another study, GABAAρ1 receptor responses were prevented by QC in a dose-dependent, fast, and reversible way. This antagonistic effect was inhibited in the existence of ascorbic acid, but not by thiol reagents changing the extracellular Cys-loop of these receptors. An amino-acid residue positioned near the ρ1 subunit GABA binding site (H141) is involved in the allosteric modulation of GABAAρ1 receptors by numerous agents such as ascorbic acid. QC likewise prevented GABA-evoked actions mediated by mutant H141DGABAAρ1 and wild-type receptors, nonetheless inhibition employed by ascorbic acid on QC actions was diminished in mutant receptors. Therefore, antagonistic actions of QC on GABAAρ1 receptors are arbitrated via a redox-independent allosteric mechanism [85].

The action of QC on the GABAA α5 receptor gene has been studied in a mouse model of KA-induced seizures. QC (50 or 100 mg/kg) treatment reduced, in a dose-dependent manner, the behavioral seizure score in mice with KA-induced seizures. Two hours after the end of the 7-d treatment regimen, GABAA α5 receptor gene expression was increased in the hippocampus by KA induction. Treatment with QC (50 mg and 100 mg/kg) reduced the KA-induced increase of GABAA α5 expression. According to these results, the expression of the GABAAα5 receptor could be a potential target of QC to reduce or to serve as a marker of seizure severity [86].

**Figure 2:**
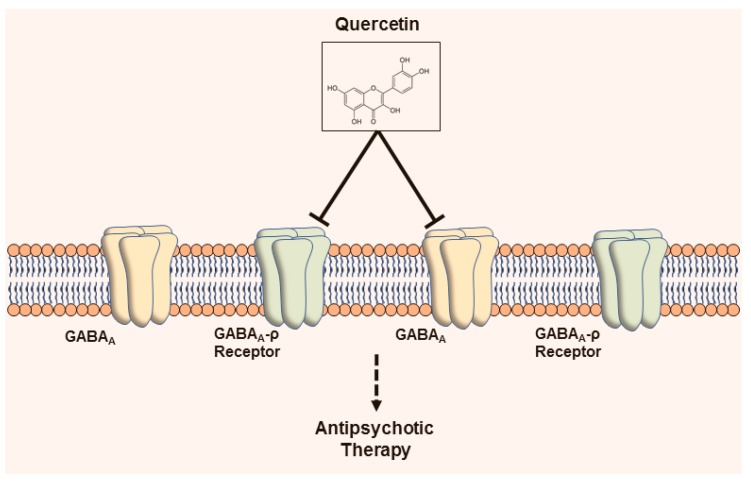
Antipsychotic activity of QC through GABA receptors. QC acts on the GABAA receptor and is possibly suitable for antipsychotic therapy.

Recently, QC has been shown to reduce prefrontal cortical GABAergic transmission and to alleviate the hyperactivity induced by the glutamatergic NMDA receptor antagonist MK-801. In cultured cortical neurons, QC noticeably decreased the GABA-activated currents in a noncompetitive manner. In mouse prefrontal cortical slices, the treatment with QC moderately prevented spontaneous and electrically evoked GABAergic inhibitory postsynaptic currents. The prefrontal-specific and systemic delivery of QC resulted in a decline in basal locomotor movement, apart from alleviating MK-801-induced hyperactivity. QC action was not fully dependent on GABAA α5, as knockdown of the α5-subunit in the prefrontal cortex elevated the MK-801-evoked psychotic symptom but reserved the QC-mediated action. Thus, QC may be a negative allosteric GABAA receptor modulator employing antipsychotic action and could be potential in the therapeutic development for psychiatric disorders [87]. QC-mediated action on GABA receptor is portrayed in Figure 2.

#### 3.2.6. Glycinergic Receptors

QC acts on the human glycine receptor alpha 1 channel expressed in Xenopus oocytes utilizing a two-electrode voltage clamp technique. In one study, it was found to reversibly inhibit glycine-induced current (I(Gly)) [88]. QC-treated inhibition depended on its dose, with an IC50 of 21.5 ± 0.2 μM, and was sensitive to membrane voltages. This QC-induced inhibition of I(Gly) was nearly eliminated upon the site-directed mutations of S267 to S267Y but not S267A, S267F, S267G, S267K, S267L, and S267T at transmembrane domain 2 (TM2). In contrast, QC increased I(Gly) in comparison with the wild-type receptor in site-directed mutant receptors including S267 to S267I, S267R, and S267V. The value of half maximal effective concentration (EC50) was 22.6 ± 1.4, 25.5 ± 4.2, and 14.5 ± 3.1 μM for S267I, S267R, and S267V, correspondingly. Therefore, QC may have the potential to regulate human glycine receptor alpha 1 via communication with amino acid residue alpha267 [88].

In cultured rat hippocampal neurons, the actions of QC on native glycine receptors (GlyRs) were examined. QC depressed glycine-induced current I(Gly) in a revocable and concentration-dependent manner, with an IC50 value is 10.7 ± 0.24 μM with a Hill coefficient of 1.08 ± 0.12. QC depressed maximum I(Gly) and suggestively altered the EC50 for glycine and the Hill coefficient. As per kinetic analysis, QC enhanced the desensitization rates. Remarkably, after the end of the glycine-with-QC co-application, a transient rebound occurred. The actions of QC also displayed voltage dependence, being greater at positive membrane potentials. Thus, QC could be a potential open channel blocker. In addition, in the sequential application protocol, QC prevented the peak amplitude of I(Gly) to a macroscopic degree while reducing GlyR desensitization. These effects implied that QC has a depressant action on the GlyR channel’s opening, which may be triggered by an allosteric mechanism. QC outstandingly prevented the recombinant-induced current mediated amplitude by alpha2, alpha2beta, alpha3, and alpha3beta GlyRs, nonetheless had no action on the alpha1 and alpha1beta GlyRs that were expressed in HEK293T cells. In addition, QC action on I(Gly) in spinal neurons during development in vitro were also studied. In spinal neurons, the degree of blockade by QC on I(Gly) was less manifested than in hippocampal neurons in a development-dependent way. Thus, QC has conceivable actions in the processing of information within a neuronal network by preventing I(Gly) and may be valuable as a pharmacological probe for recognizing the subunit types of GlyRs [89].

### 3.3. Miscellaneous Targets

#### 3.3.1. Toll-Like Receptors and Cytokine Receptors

In several studies, QC produces its anti-inflammatory activity by acting on TLR4 and cytokines. In LPS-triggered signaling via TLR4, QC suppresses the nuclear factor of kappa light polypeptide gene enhancer in B degradation, with subsequent activation of nuclear factor-kappa B (NF-κB) as well as activation of phosphorylation of p38 and Akt in bone marrow-derived macrophages. In tumor necrosis factor-α (TNF-α)-induced signaling, QC significantly repressed the interleukin (IL)-6 production and NF-κB activation [90].

QC protected against LPS-induced expressions of cell surface molecules such as cluster of differentiation (CD)80, CD86, and major histocompatibility complex class I/II and pro-inflammatory cytokines such as IL-1β, TNF-α, IL-6 and IL-12p70 but protecting action was inhibited toll-interacting protein silencing in RAW264.7 cells. In addition, QC treatment inhibited LPS-induced activation of mitogen-activated protein kinase (MAPK), including p38, c-Jun N-terminal kinase (JNK), and ERK1/2 and the NF-κB (p65) translocations via toll-interacting proteins. QC treatment also displayed a significant reduction in prostaglandin E2 and cyclooxygenase-2 levels as well as inducible nitric oxide synthase (iNOS)-mediated production of nitric oxide (NO) production by LPS induction [91].

The treatment of QC prevented the NF-κB nuclear translocation and cytokine release. In addition, QC prevents the release of TNF-α by acting on the NF-κB signaling pathway. Additionally, oxidized low-density lipoprotein-induced inflammation was also connected to the p38MAPK, ERK1/2, and JNK, and Akt pathway activations, and the QC-mediated action may also be related to protecting from the activation of these pathways. In addition, QC suggestively downregulated the increase TLRs and TNF-α expression at mRNA level in high carbohydrate diet-fed atherosclerotic rats. As QC displays a preventive action on the TLR-mediated MAPK and NF-κB signaling pathways, it could be a potential agent in the protection and management of atherosclerosis by decreasing detrimental vascular inflammation [92].

QC displayed anti-inflammatory activity in BV-2 microglial cells. It pointedly prevented LPS-induced NO release and expression of iNOS. QC notably prevented NF-κB activation by protecting from the degradation of nuclear factor of kappa light polypeptide gene enhancer in B-cells inhibitor, alpha [93]. QC also showed anti-inflammatory effects in another study conducted on BV-2 microglial cells, where QC was 10-fold more potent than cyanidin in the inhibition of LPS-induced NO release [94].

#### 3.3.2. Neurotrophic Factors

Several studies have reported the activity of QC on neurotrophic factors. Brain-derived neurotrophic factor (BDNF) is a vital neurotrophin that plays an important role in the survival of neuronal cells. In a recent study, QC (20 and 50 mg/kg) significantly enhanced the mRNA BDNF expression compared with that of a control group and produced neuroprotective effects [95]. In addition, QC derived from *Ginkgo biloba* extract (EGb 761) stimulates depression-related signaling pathways involving BDNF/phosphorylation of CREB/postsynaptic density proteins-95 [96]. QC declines Aβ in neurons collected from the double transgenic AD mice (TgAPPswe/PS1e9). The administration of QC increases BDNF expression and reduces Aβ oligomers in the hippocampus of the TgAPPswe/PS1e9, which correlated with mouse cognitive improvement [96]. In another study, QC treatment reduced cell apoptosis in the focal cerebral ischemia rat brain in a way of a mechanism that may be related to the activation of the BDNF–tropomyosin receptor kinase B–phosphoinositide 3-kinases/Akt signaling pathway [97]. QC meaningfully activated the Akt and 3-phosphoinositide-dependent protein kinase 1 (PDK1) in MN9D dopaminergic neuronal cell lines. Regarding the blocking or siRNA, knockdown of PDK1 prevented the Akt activation. Therefore, Akt is a downstream signaling protein of PDK1 in the QC-mediated neuroprotection. QC also enhanced CREB phosphorylation and elevated the mitochondrial bioenergetics ability and prevented 6-hydroxydopamine-intoxicated toxicity in MN9D cells. In a MitoPark transgenic mouse model of PD, the attenuation of cognitive deficits, depletion of striatal dopamine and TH neuronal cell loss were accompanied by QC administration [8].

In PC12 cells, QC dose-dependently stimulated nerve growth factor (NGF)-induced neurite outgrowth. QC-mediated stimulatory action was abolished by the knockdown of Na-K-Cl cotransporter (NKCC1) via RNAi methods; QC stimulated NKCC1 activity without any elevation in the NKCC1 protein expression. The action of QC on neurite outgrowth was dependent on extracellular Cl-. Thus, QC stimulates NGF-induced neurite outgrowth via increasing Cl- incorporation into the intracellular space by stimulating NKCC1 [98]. Furthermore, QC, like the extract of *Ginkgo folium*, could enhance the effect of NGF in cultured PC12 cells. QC potentiates neurite outgrowth and phosphorylation of ERK1/2 [99].

**Figure 3:**
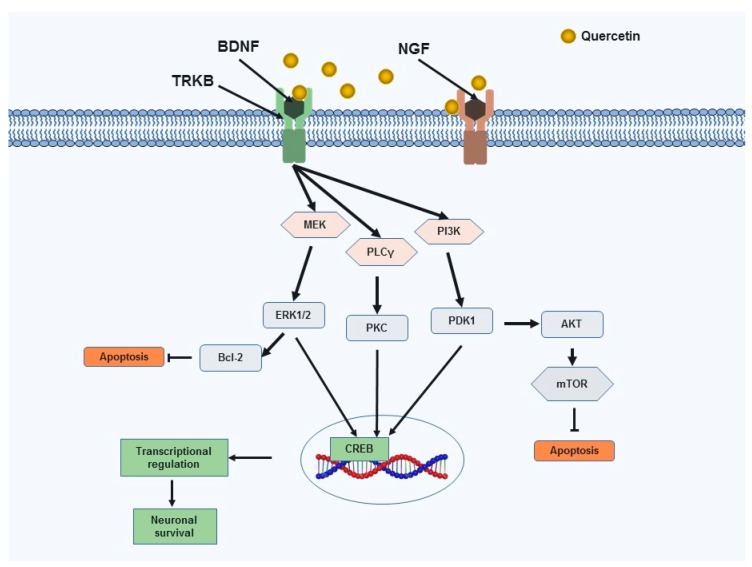
Molecular pathways regarding neuronal survival on which QC seems to assess their effects within the brain. QC-mediated action on neurotrophic factors BDNF and NGF. QC activates BDNF-TrkB and its associated signaling pathway, which ultimately, results in phosphorylation of CREB, and the CREB-mediated neuronal survival effect [101]. BDNF: Brain-derived neurotrophic factor; TRKB: Tropomyosin receptor kinase B; NGF: Nerve growth factor; PLCγ: Phospholipase C gamma; PI3K: Phosphatidylinositol 3-kinase; PKC: Protein kinase C; ERK: Extracellular signal-regulated kinase 1/2; PDK1: 3-phosphoinositide-dependent protein kinase 1; mTOR: Mammalian target of rapamycin; Akt: Protein Kinase B; Bcl-2: B-cell lymphoma 2; CREB: Cyclic AMP response element binding protein.

QC protects against neurodegeneration in a model of diabetic retinopathy [100]. Treatment with QC suggestively elevated the neurotrophic factors (BDNF, NGF) and prevented a rise in caspase-3 activity and cytochrome c level in the diabetic retina. In addition, the expression of B-cell lymphoma 2 (Bcl-2) was increased in the QC-treated diabetic retina. Therefore, QC may have the pharmacological potential to protect against neuronal damage in the diabetic retina by attenuating the expression of neurotrophic factors and correspondingly by preventing the neuronal apoptosis [100]. QC-mediated action on neurotrophic signaling leading to neuronal survival and protection are portrayed in Figure 3.

#### 3.3.3. Apolipoprotein E

Apolipoprotein E (APOE) is encoded by the APOE gene which consists of 299 amino acid proteins. Change of a single amino acid of the APOE protein is due to the three common polymorphisms in the APOE gene—ɛ2, ɛ3, and ɛ4. With the change to the APOE ɛ2, ɛ3, and ɛ4 alleles, the likelihood of evolving cerebral amyloid angiopathy and AD is greater. This relationship is observed in a dose-dependent manner. APOE ɛ4 is mainly connected to an elevated risk for AD, whereas APOE ɛ2 is associated with a decreased risk. The actions of the APOE genotype on the risk of these disorders are likely to be arbitrated by differential properties of APOE on the accumulation of Aβ in the brain and its vasculature. Interestingly, the response pattern to AD treatment might differ with APOE genotype [102]. QC-enriched diets induced hepatic paraoxonase/arylesterase 1 gene expression, with a propensity for superior induction in APOE ɛ3 in comparison with APOE ɛ4 mice. In addition, hepatic mRNA and protein levels of β-glucuronidase and sulfatase, both enzymes centrally connected to the deconjugation of QC conjugates, were lesser in APOE ɛ4 in comparison with APOE ɛ3 mice. Peroxisome proliferator-activated receptor gamma (which partially controls the expression of the paraoxonase/arylesterase 1 gene) mRNA levels were lesser in APOE ɛ4 in comparison with APOE ɛ3 mice [103].

QC displayed blood pressure-lowering actions in overweight/obese carriers of the APOE ɛ3/APOE ɛ4 genotype but not in carriers of the ɛ4 allele [104]. Elevated APOE in the brain may be an operative therapeutic approach for AD. QC can also meaningfully increase APOE levels by hindering APOE degradation in immortalized astrocytes. In the 5xFAD mouse model, QC significantly elevated brain APOE, and declined insoluble Aβ levels in the cortex. Therefore, QC increases APOE levels via a novel mechanism and may be developed as a novel class of drug for AD therapy [105]. More research should be designed to reveal the in-depth mechanism of QC on APOE.

#### 3.3.4. Nuclear Factor Erythroid 2-Related Factor 2-Antioxidant Responsive Element

In recent times, the antioxidant activity of QC focusing on the Nrf2-ARE pathway have been addressed by several investigations in the models of neurological disorders. QC protected PC12 cells from 1-methyl-4-phenylpyridinium-induced oxidative stress and degeneration by Nrf2-mediated upregulation of the heme oxygenase-1 (HO-1), NQO1 and glutathione [106]. QC also protected against high-glucose-induced oxidative stress in SH-SY5Y cells. Under the chronic high-glucose conditions, it enhanced Glo-1 functions in central neurons which may be mediated by activation of the Nrf2/ARE pathway. In addition, QC-treated PKC activation increased phosphorylation of Nrf2. Moreover, glycogen synthase kinase-3β inhibition may be connected to the QC-mediated Nrf2/ARE pathway activation [107].

In LPS-induced murine BV-2 microglial cells, QC produced a greater stimulating effect on Nrf2-induced increase expression of heme-oxygenase-1 (HO-1) protein than cyanidin. QC upregulated Nrf2/HO-1 activity in terms of endotoxic stress. QC upregulated HO-1 against endotoxic stress via the participation of MAPKs [94].

**Figure 4:**
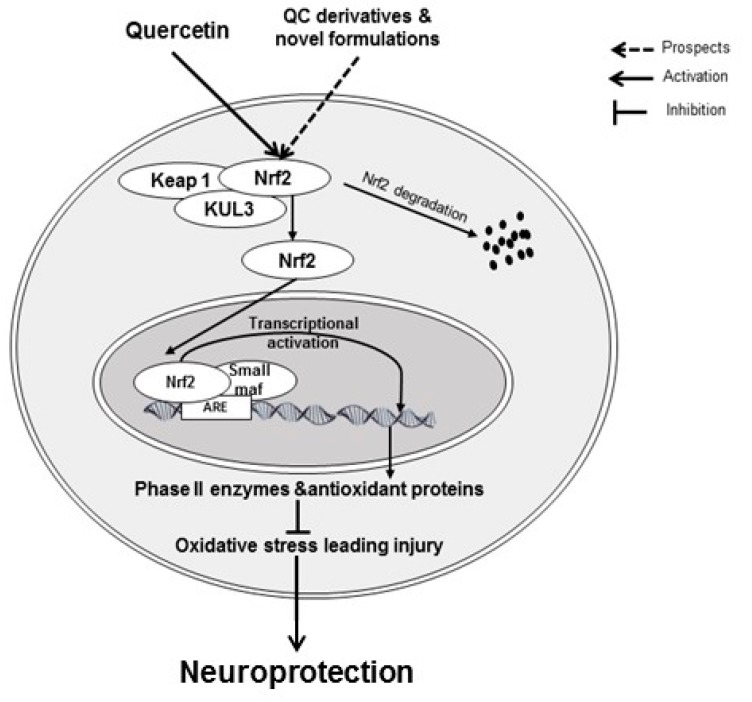
QC prevents oxidative stress leading to damage by activating the Nrf2 pathway. QC-mediated Nrf2 activation leads to transcriptional activation of antioxidant response elements. This activation results in prevention against oxidative damage and ultimately leads to amelioration of cognitive impairment. Keap1: Kelch-like ECH-associated protein 1; Nrf2-ARE: Nuclear factor erythroid 2.

QC displayed neuroprotective activity in manganese-, domoic acid- and d-galactose-induced neurotoxicity models [108,109,110]. In a model of Mn-intoxicated inflammatory and apoptosis response in SK-N-MC cells and Sprague Dawley rats, QC-mediated protective actions that may be connected to the stimulation of HO-1/Nrf2 and prevention of the NF-κB pathway [108]. In domoic acid-intoxicated memory impairment, QC treatment activated Nrf2-ARE and decreased protein carbonylation and reactive oxygen species in mice. Moreover, the activity of AMP-activated protein kinase (AMPK) was suggestively elevated in the QC-treated group [109]. QC also inhibited changes in the cell morphology and apoptosis in the hippocampus along with elevated the expression of Nrf2, HO-1, and superoxidase dismutase in D-galactose-induced mice. Brusatol (a Nrf2 inhibitor) treatment reversed the QC-mediated HO-1 and superoxidase dismutase expression and protection of cells [110]. The action of QC on Nf2-ARE signaling pathway leading to neuroprotection is displayed in Figure 4.

#### 3.3.5. Sirtuins

In the numerous molecular pathways, seven types of sirtuins (SIRT1 to SIRT7) are involved in diversity, with different cellular localization and molecular targets in mammals [111]. Of these, sirtuin 1 (SIRT1) mostly localizes in the nucleus and acts as a deacetylase for histones and other targets. SIRT1 protects cells from apoptosis and promotes the differentiation of stem cells. SIRT2 is prevalent in the cytoplasm and has been found to accumulate in neurons, while other SIRTs localize primarily in the mitochondria [111]. Sirtuin 6 (SIRT6) is crucial in regulating various cellular processes such as glucose metabolism and genomic stability [112]. Sirtuins has physiological role on the progression of NDDs by modulating transcriptional activity along with directly deacetylating proteotoxic species. Targeting sirtuin proteins are crucial in finding nonprotective agents for several NDDs such as PD, Huntington’s disease, AD, spinal and bulbar muscular atrophy, and amyotrophic lateral sclerosis [113,114,115].

**Figure 5:**
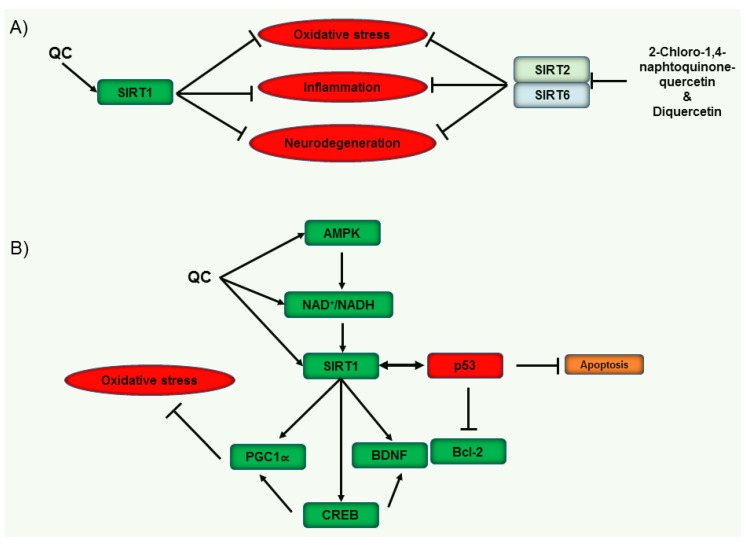
QC-mediated regulation of sirtuins for the therapy of cognitive impairment. The action of QC on several sirtuin proteins, leads to protective action. (**A**) QC-mediated activation of SIRT1 and 2-chloro-1,4-naphtoquinone-quercetin- and diquercetin-mediated prevention may have potential in the prevention of oxidative stress, inflammation and neurodegeneration. (**B**) QC-mediated stimulation of SIRT1 and regulation of AMPK and NAD+/NADH may have potential against oxidative stress and apoptosis. QC: Quercetin; SIRT: Sirtuin; AMPK: AMP-activated protein kinase; NAD: nicotinamide adenine dinucleotide; BDNF: Brain-derived neurotrophic factor; Bcl-2: B-cell lymphoma 2; CREB: Cyclic AMP response element binding protein.

As per several studies, QC has actions on SIRT1, and in an investigation, the beneficial action of QC on lipid and glucose metabolism disorder were connected to the upregulation of SIRT1 expression and its impact on the Akt signaling pathway [116]. In another study, QC suppresses oxidized low-density lipoprotein-induced endothelial oxidative damages by regulating the NADPH oxidase/AMPK/Akt/endothelial nitric oxide synthase signaling pathway and stimulating SIRT1 action [117]. In addition, QC might suppress adipose tissue macrophage inflammation and infiltration through the AMPKα1/SIRT1 pathway in high-fat diet-fed mice [118]. A recent study addressed the neuroprotective action of QC via the SIRT1-mediated pathway, where QC plays a crucial role against excitotoxic neurodegeneration which is potential for the therapy of motor neuron disorders [119]. QC displayed the highest antioxidant activity compared to other tested polyphenols in an in vitro comparative study. Polyphenols can improve the expression of SIRT1 as well as the activation of AMPK [120]. In an investigation on QC derivatives, diquercetin and 2-chloro-1,4-naphtoquinone-quercetin were recognized as auspicious SIRT6 inhibitors with IC50 130 μM and 55 μM, respectively. 2-Chloro-1,4-naphtoquinone-quercetin inhibited SIRT2 (IC50: 14 μM). The Michaelis constant (Km) value of nicotinamide adenine dinucleotide (NAD+) are elevated by diquercetin, while 2-chloro-1,4-naphthoquinone-quercetin elevated the Km value of the acetylated substrate. The binding site of the nicotinamide moiety are preferred by diquercetin, but 2-chloro-1,4-naphtoquinone-quercetin docked with the substrate binding site as per molecular docking studies. Overall, diquercetin competes with NAD+, while 2-chloro-1,4-naphthoquinone-quercetin competes with the SIRT6 (acetylated substrate in the catalytic site) [112]. QC actions on sirtuin proteins are presented in Figure 5.

## 4. Closing Remarks

We have recently published that the methanol extract of red onion displays anti-inflammatory activity against LPS-induced BV-2 microglial cells via preventing several inflammatory markers. In addition, the extract also upregulated anti-apoptotic markers and several antioxidant enzymes in N27-A cells [19]. Considering the data, we are interested in QC, which is one of the major components of red onion extract. As a phytochemical constituent, QC has demonstrated its pharmacological role in the models of several chronic diseases and disorders. Several recent studies have proved its potential therapeutic activity in neurological disorders. In our discussion, we present its role in modulating several signals focusing on neurodegenerative diseases. The role of QC on sodium and calcium channel signaling may have the potential to protect against neuronal damage in neurodegenerative diseases. QC also acts on several molecules in the dopaminergic signal pathway that may be helpful in basic research on disorders such as PD, schizophrenia, and attention deficit hyperactivity disorder. QC is a potentially bioactive compound against cognitive deficits, as it shows a role in acetylcholinergic, serotonergic, and glycinergic systems. Further study on these systems may help find the molecular role of QC in preventing cognitive impairment.

The action of QC on TLR-4 and cytokine signals may indicate its potential role against several neuroinflammatory conditions, as toll-like and cytokine receptors are considered crucial targets to the discovery of anti-inflammatory agents to treat neuroinflammatory conditions. To date, the role of QC has only been studied on TLR-4; investigations into its role in other TLRs should be conducted.

An essential action of QC is on Nrf2-ARE signals. Further studies should be designed to correlate these signals with other receptors. QC also produces suggestive effects on several neurotrophic factors, effects that range from neuronal survival to neuronal protection. Additionally, QC and its derivatives have actions on sirtuins. The QC-mediated activation of SIRT1 and it-mediated inhibition of SIRT6 may be potential in neuroprotective therapy. It would be fascinating to study the expression patterns of these neurotrophic factors after knockdown and knockout of potential receptors.

Finally, the ability of QC to modulate several drug targets may be an attractive research focus in the quest to discover treatments for neurodegenerative diseases. Some of this research has already been conducted, and, according to recent studies, the role of QC in nuclear receptors, (e.g., estrogen receptors and peroxisome proliferator-activated receptors) should be investigated. While GPCRs are considered crucial targets in the treatment of neurological disorders, QC’s role in numerous GPCRs such as metabotropic glutamate, cannabinoid, and opioid receptors remains to be studied. Thus, a target-based study of QC and its analogs may help establish potential therapies for the treatment of several neurodegenerative conditions.

QC has actions on multiple targets; however, it cannot cross the BBB due to its water insolubility and low oral bioavailability, a major stumbling block in central nervous system therapeutics. Several novel deliveries of QC have been conducted to enhance its bioavailability. QC nanoparticle and nano emulsion protected ischemia [121,122] and QC nanoparticles prevented neuroinflammation in rodent models [123]. The study of novel deliveries of QC and its derivatives on several receptors for neuroprotective therapy would be interesting areas of research. In addition, the designing of QC derivatives considering the pharmacokinetic limitations would also be fascinating for the therapy of cognitive impairment. Another important area of research would be conducting studies of QC, its derivatives and novel formulations on patients with cognitive impairment at the clinical level. Preclinical studies are needed to find its potential derivatives and novel formulations.

## Figures and Tables

**Table 1:** Several derivatives of QC with their potential targets for neuroprotective therapy.

Sl	Common Name	Substituents							Sources	Targets in Neuroprotection	Ability to crossBBB	References
		R1	R2	R3	R4	R5	R6	R7				
1	Quercetin 3′-O-(3-chloropivaloyl) (quercetin pivaloyl ester)	OH	OH	H	OH	H	OH	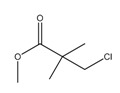	Synthetically modified quercetin	Toll-like receptor (TLR)-4	+	[43]
2	3, 5, 7, 3’, 4’-pentahydroxyflavon (quercetin)	OH	OH	H	OH	OH	OH	H	Elderberries, cranberries, coriander leaves, canned capers	Nuclear factor erythroid 2-related factor 2-antioxidant responsive element (Nrf2-ARE) and antioxidant/anti-inflammatory enzyme paraoxonase 2 (PON2) enzyme	+	[13,44]
3	Quercetin 3-O-glucoside (isoquercetin)	O-Glc	OH	H	OH	OH	OH	H	Mango fruits, beans, plums, onions	TLR-2 and 4	+	[44,45,46,47]
4	Quercetin 3-O-rhamnoside (quercitrin)	O-Rha	OH	H	OH	OH	OH	H	Mango–fruits, pepper–fruits, cranberry, lingonberry	TLR-2 and 4	−	[28,45,47]
5	Quercetin 3-O-rhamnosyl-(1–6)-glucoside (rutin)	O-X	OH	H	OH	OH	OH	H	Plums, cherries, tomatoes, buckwheat leaves, buckwheat seeds, chokeberry	−	+	[48,49,50]
6	Quercetin 7-O-glucoside	OH	OH	H	O-Glc	OH	OH	H	Beans	−	−	[23]
7	Quercetin 3-O-rhamnoside-7-O-glucoside	O-Y	OH	H	Glc	OH	OH	H	Pepper fruits	−	−	[28]
8	Quercetin 3-sulfate-7-O-arabinoside	O-Sul	OH	H	O-Ara	OH	OH	H	Salt bush	−	−	[21]
9	Quercetin 3-O-glucoside-3’-sulfate	O-Glc	OH	H	OH	O-Sul	OH	H	Corn flower	TLR-2 and 4	−	[47,51]
10	Quercetin 5-methyl ether (azaleatin)	OH	O-M	H	OH	OH	OH	H	Flowers of *Rhododendron mucronatum*, *Plumbago capensis*, *Ceratostigmawillmottiana*, *Carya pecan*, leaves of *Eucryphia*	Extracellular signal-regulated kinase 1/2 (ERK)-pathway	−	[13,52]
11	Quercetin 7-methyl ether (rhamnetin)	OH	OH	H	O-M	OH	OH	H	Cloves, berries from buckthorn family, such as *Rhamnusinfectorius, R. cathartica*	−	+	[13,53]
12	Quercetin 3’-methyl ether (isohramnetin)	OH	OH	H	OH	O-M	OH	H	Honey	TLR-2 and 4	+	[47,54,55]
13	Quercetin 4’-methyl ether (tamarixetin)	OH	OH	H	OH	OH	O-M	H	*Artemisia annua*	TLR-4 and myeloid differentiation primary response 88	+	[13,55,56]
14	Quercetin 7-methoxy-3-O-glucoside	O-Glc	OH	H	O-M	OH	OH	H	Honey	−	−	[54]
15	Quercetin 3’-methoxy-3-O-galactoside	O-Gal	OH	H	OH	O-M	OH	H	Sage	–	−	[24]
16	6, 5’-Di-C-prenylquercetin	OH	OH	Z	OH	OH	OH	Z	Paper mullberry	−	−	[57]
17	Quercetin-3-O-glucuronide	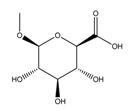	OH	H	OH	H	OH	OH	Red wine	Cyclic AMP response element binding protein (CREB) phosphorylation and ↓amyloid beta (Aβ)_1-40_	+	[58,59]
18	7-O-galloylquercetin	OH	OH	H	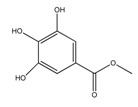	H	OH	OH	Semisynthetic flavonoid	Activate Nrf2/ARE and ↑antioxidant enzyme NAD(P)H quinone oxidoreducase-1 (NQO1)	−	[60]

## Abbreviations

6-OHDA	6-hydroxydopamine
AD	Alzheimer’s disease
Akt	Protein Kinase B
AMPA	α-amino-3-hydroxy-5-methyl-4-isoxazolepropionic acid
APOE	Apolipoprotein E
Aβ	Amyloid beta
BBB	Blood-brain barrier
Bcl-2	B-cell lymphoma 2
BDNF	Brain-derived neurotrophic factor
CD	Cluster of differentiation
CREB	Cyclic AMP response element binding protein
ERK	Extracellular signal-regulated kinase 1/2
GABA	Gamma-aminobutyric acid
IACh	Inward currents
IL	Interleukin
iNOS	Inducible nitric oxide synthase
Keap1	Kelch-like ECH-associated protein 1
MAPK	Mitogen-activated protein kinase
MPTP	1-methyl-4-phenyl-1,2,3,6-tetrahydropyridine
mTOR	Mammalian target of rapamycin
NDDs	Neurodegenerative diseases
NF-κB	Nuclear factor-kappa B
NGF	Nerve growth factor
NKCC1	Na-K-Cl cotransporter
NMDA	N-methyl-D-aspartate
NO	Nitric oxide
NQO1	NAD(P)H quinone oxidoreducase-1
Nrf2-ARE	Nuclear factor erythroid 2-related factor 2-antioxidant responsive element
PD	Parkinson’s disease
PDK1	3-phosphoinositide-dependent protein kinase 1
PI3K	Phosphatidylinositol 3-kinase
PKC	Protein kinase C
PLCγ	Phospholipase C gamma
QC	Quercetin
SIRT	Sirtuin
TLR	Toll-like receptor
TrkB	Tropomyosin receptor kinase B
α7 nAChR	α7 nicotinic acetylcholine receptor
HO-1	Heme-oxygenase-1
